# Organising a Safe Space for Navigating Social-Ecological Transformations to Sustainability

**DOI:** 10.3390/ijerph120606027

**Published:** 2015-05-28

**Authors:** Laura Pereira, Timothy Karpouzoglou, Samir Doshi, Niki Frantzeskaki

**Affiliations:** 1 School of Environmental and Geographical Sciences, University of Cape Town, Private Bag X3, Rondebosch 7701, South Africa; 2 Public Administration and Policy Group, Wageningen University, Hollandseweg 1, Wageningen 6700 EW, The Netherlands; E-Mail: timothy.karpouzoglou@wur.nl; 3 American Association for the Advancement of Science, 1200 New York Ave NW, Washington, DC 20005, USA; E-Mail: sdoshi@uvm.edu; 4 Dutch Research Institute for Transitions, Faculty of Social Sciences, Erasmus University, Rotterdam 3062 PA, The Netherlands; E-Mail: n.frantzeskaki@drift.eur.nl

**Keywords:** sustainability, safe space, resilience, transitions, complexity, transformation, learning, reflexivity, knowledge co-production

## Abstract

The need for developing socially just living conditions for the world’s growing population whilst keeping human societies within a ‘safe operating space’ has become a modern imperative. This requires transformative changes in the dominant social norms, behaviours, governance and management regimes that guide human responses in areas such as urban ecology, public health, resource security (e.g., food, water, energy access), economic development and biodiversity conservation. However, such systemic transformations necessitate experimentation in public arenas of exchange and a deepening of processes that can widen multi-stakeholder learning. We argue that there is an emergent potential in bridging the sustainability transitions and resilience approaches to create new scientific capacity that can support large-scale social-ecological transformations (SETs) to sustainability globally, not just in the West. In this article, we elucidate a set of guiding principles for the design of a ‘safe space’ to encourage stronger interactions between these research areas and others that are relevant to the challenges faced. We envisage new opportunities for transdisciplinary collaboration that will develop an adaptive and evolving community of practice. In particular, we emphasise the great opportunity for engaging with the role of emerging economies in facilitating safe space experimentation.

## 1. Introduction

Humanity faces a daunting challenge in the 21st century: to improve human wellbeing and reduce economic inequality globally whilst remaining within ecological ‘planetary boundaries’. The past two centuries have seen dramatic changes in how humans interact both with each other and with the Earth’s ecosystems that are relied upon for survival and prosperity. In essence, this increase in what we have come to describe as human wellbeing has largely been achieved through the technological progress spearheaded by the industrial revolution, which coincided with the large-scale conversion of land to agriculture, the appropriation of natural resources and the settlement of burgeoning populations in urban areas. At the same time, progress around innovations in public health medicines and measures to reduce mortality and morbidity rates have also accelerated under the capitalist-industrialist paradigm. The extent and cumulative impact of the world’s current development pathway now substantially affects the sustainability of ecosystems and potentially threatens human societies [1]. Under what has been termed the ‘Great Acceleration’, the impact of the human enterprise on the Earth system from the start of the industrial revolution onwards has been unprecedented [2]. The scale of human changes to the Earth have come to rival native ecological factors and have inadvertently forced us into a new planetary era—defined as the Anthropocene [3,4]. With this in mind, there is a growing recognition that dramatic socio-cultural, political and technological changes are required to transform human societies towards a ‘Good Anthropocene’ paradigm—a future that meets the deeply intertwined development and environmental challenges society faces, in a world profoundly shaped by human actions [5]. These fundamental shifts in human-environmental relationships are referred to as ‘social-ecological transformations’ (SETs) and aim to create opportunities for societal wellbeing today and in the future, while strengthening the Earth’s life support systems [6].

Understanding how the Earth system and human society function as a complex adaptive system requires us to use different language, referring to emerging and navigating transformations over managing, steering, or controlling transitions [7]. The implications are potentially vast. Maintaining human development within planetary boundaries [1] while improving societal wellbeing will invariably require both disruptive innovations and systemic transformations that address the root causes of these issues in the dominant social norms, behaviours and practices [8].

At the same time it requires us to think more critically and with greater rigor about “What will such radical and systemic SETs to sustainability look like? How can research become re-aligned and co-developed by academic communities, civil society, policy and business so as to inform and inspire solutions to real-world problems?” Addressing these types of questions necessitates far greater experimentation and the development of deeper engagement with questions of sustainability, where cognitive and experiential diversity as well as a deliberate environment for knowledge exchange is crucial [9].

We draw our main insights from focusing on key trends and developments in two important interdisciplinary research strands that have come of age in transformations research—these are sustainability transitions and resilience approaches. We will leverage these two perspectives as examples of research approaches that are engaging with the issue of transformations (Other examples of research fields that have taken an active interest in transformations research include social innovation and entrepreneurship studies, sustainability science and development studies) to explore how a safe space could become a vehicle for inspiring new research ideas and collaborations.

Studies on knowledge creation have emphasized how cognitive diversity—the extent to which differences in knowledge, beliefs, preferences and perspectives are held—is critical to the emergence of new knowledge whether it be amongst different disciplines or different sectors, such as academics and practitioners [10]. This is what we refer to as a form of “bridging” of topic areas to harness a new trans-paradigmatic way of knowledge production similar to that emphasized by transdisciplinarity, but that goes beyond an emphasis on methodologies that integrate multiple perspectives towards a more emergent co-production. Such an endeavour has to be societally relevant, impact orientated and go beyond merely a simple exercise of combining different conceptual perspectives. However, the interaction and integration process that facilitates this knowledge co-production requires communication that reflects open-mindedness norms, which not only encourage the expression of different views but also values and utilizes others’ knowledge and ideas [11]. These norms are the driving principles behind a safe space. We further wish to highlight the critical need for engaging with the role of emerging economies in enacting social-ecological transformations, specifically the BRICS countries (Brazil, Russia, India, China and South Africa), in facilitating a safe space as thus far much of the work in the sustainability space has occurred in Western contexts, driven by Western research programmes.

The paper structure is as follows: In the first part of the article we describe the contributions of resilience and transitions research to the understanding of sustainability transformations. We highlight particular project initiatives that have been developed within these research communities as well as explore some of the main differences in terms of epistemological and ontological starting points. We also describe challenges and opportunities for sustainability transformations research in emerging economies. In the second section of the article, we elaborate what we mean by a safe space and expand on a set of guiding principles that can guide and broaden multi-stakeholder learning and collaboration in the safe space. We further argue why deepening engagement with contexts of inequality is relevant so that the safe space becomes relevant for addressing real life problems across a broader set of stakeholders in both Northern and Southern settings. We conclude by providing a summary of key insights of the article as well as direct attention to potential areas of caution in the enactment of the proposed safe space.

## 2. Emerging Perspectives on Sustainability Transformations

### 2.1. Resilience Perspectives on Transformations

For the past two decades, a growing group of resilience scholars have been studying transformations toward improved ecosystem stewardship and global sustainability [6,12]. Most of these studies have been carried out at local to national levels, but in light of the recent work in Earth system science and on planetary boundaries [13], the need for radical transformations in human-environmental interactions and feedbacks to reverse current trends of crossing critical thresholds and tipping points in the whole Earth system have also been discussed [14]. This literature underpins the need for social-ecological transformations (SETs), which are described as ‘untried beginnings from which to evolve a fundamentally new way of living when existing ecological, economic and social conditions make the current system untenable’ [6].

Resilience scholars note the important aspect of transformability, understood as the capacity to cross thresholds into new development trajectories, in smaller social-ecological systems, so as to maintain the resilience of the Earth system as a whole [15]. The capacity of a system to transform at smaller scales draws on resilience from multiple scales, making use of crises as windows of opportunity for novelty and innovation, and recombining sources of experience and knowledge to navigate these social–ecological transitions [15]. In their work, resilience scholars draw on a range of community-led solutions, business innovations and governance experiments that aim to foster large-scale sustainability transformations, which they refer to as social-ecological innovations [16]. Examples of such innovations include clean energy financing mechanisms (e.g., the Asian Development Bank’s Clean Energy Financing Partnership Facility: http://www.adb.org/site/funds/funds/clean-energy-financing-partnership-facility), Resilience Innovation Labs (RILabs) addressing community health issues, food sovereignty movements using agroecological farming practices (The Cuban Campesino-to-Campesino agroecology movement: http://www.campesinocubano.anap.cu/, see [17]), and more. This strand of resilience research is in part inspired from the work of the University of Waterloo’s Institute for Social Innovation and Resilience. It is in this school of thought that the links between socio-ecological innovation and transformation have been put forward more explicitly [18,19,20,21]. Within this emerging research field particular attention is placed on ‘processes’ of social learning, innovation scaling-up that addresses systemic barriers and scaling-out to increase adoption, and the particular importance of human agency traced down to individuals and social groups within organisations [20]. Innovation in particular is understood to have a crucial role in enabling SETs since it provides leverage and opportunity for steering away from potentially critical thresholds in the earth system and opens up new trajectories of sustainability [16,22].

### 2.2. Transitions: An Experimentation Lens for Enabling Transformations

Transition governance has been developed as an approach to create and understand the conditions and context under which co-evolutionary processes are more likely to lead to sustainable outcomes. Almost in parallel to the development of transformations scholarship under resilience thinking, transition management and socio-technological transitions studies have been pioneered by European researchers [23,24,25,26,27]. A growing community of science, technology and society (STS) scholars characterise sustainability transitions as non-linear, co-evolutionary processes of structural systemic change between technologies (including infrastructures), institutions, and networks of producers, consumers, intermediaries, and regulators [28]. Transition governance is developed as an approach to create conditions and context under which such co-evolutionary processes are more likely to lead to sustainable outcomes and might be triggered and supported [29].

In transition studies and especially in transition management, experimentation has been conceptualized and empirically proven to be a driving force for supporting and enabling larger and long-term transformations. In all the different schools of transition studies, experimentation has been examined: how historically it creates co-evolving transformation pathways [26], how alternative practices can be nurtured in experimental settings to be shielded from competitive dominant regimes [29,30], and how experiments can be formulated and realized as stepping stones for the more radical transformative agendas’ actualisation [31,32,33,34,35,36].

A series of productive research collaborations are now underway within Europe that signify a coming of age of a new type of systemic integrated approach to researching global sustainability challenges (such programmes include the Transformative Social Innovation Theory (TRANSIT) initiative, the Accelerating and Rescaling Transitions to Sustainability (ARTS) initiative and the High-end climate change and integrative solutions (IMPRESSIONS) programme). All these research programmes have moved beyond the technological lens of previous transitions work to bring new theoretical and empirical grounds to the sustainability transitions field by addressing issues like the politics of sustainability transitions, multi-actor governance and cross-scale dynamics of sustainability transitions from an agency perspective. This turn to a relational approach, see [37] to examine contemporary sustainability transitions and their dynamics comes from a need to understand politics and multi-level governance patterns and possibilities better; characteristics of sustainability transitions have so far only been researched to a limited extent [38,39,40,41].

### 2.3. Grey Areas of Epistemological and Ontological Nature

Ontological and epistemological differences can have a profound effect on the framing and boundary-setting of sustainability paradigms [42]. Arguably epistemological and ontological differences can also be a source of tension and dispute. For example, transitions thinkers have often criticised resilience thinkers for not adequately taking into account issues of power and inequality [25]. The role of power is more pronounced in innovation and transition management to analyze both the structural power of regimes to sustain their position as well as innovative power to transform regimes [43,44]. Equally, the resilience school of thought has raised some concerns about transitions not taking into account environmental limits.

This area of divergence perhaps also marks an area of future opportunity where stronger links between the two approaches become both possible and desirable. According to Olsson *et al*. [7], one of the important challenges for enacting sustainability transformations is to focus more on emerging technologies and social innovations, and the exploration of the features of institutional settings that allow for novelty, fail-safe experimentation, and continuous learning, but also equally account for innovations that may carry social-ecological risk [45]. For example, the NeWater project, has combined insights from resilience thinking and transition management to understand large-scale water management transitions [35]. Insights have included a wide range of focal areas, including adaptive management [46], policy change [47], the role of pilot projects [48], experimentation [49] and strategy [50]. As we look to scale-up certain test cases of sustainability innovations, a need for an evidence base of which innovations can be successful at scale and have the most impact is needed and this includes a need to look holistically at the enabling environment (policy, strategy, buy-in *etc*.) for these innovations to thrive. The fields of development economics and impact evaluation have produced various rigorous experimental and non-experimental methods that aggregate quantitative and qualitative data to determine causal inference of a specific innovation and its impact on the social-ecological system [51]. In order to provide real-life impact, it will be necessary to draw learning from these areas into the resilience and transitions approaches so as to be able to provide a language of rigour in fields that are often quite nebulous.

**Table 1:** Outlines how elements of both resilience and transitions frameworks may be combined to counter their respective weaknesses.

Potential for Understanding	Resilience	Study Example and Country of First Author	Transitions	Study Example and Country of First Author
Transformations towards improved ecosystem stewardship	Strong	Westley *et al.* 2011 [6] (Canada)	Weak	Loorbach 2010 [23] (The Netherlands)
System transformation through cross scale interaction	Strong	Folke *et al*. 2010 [52] (Sweden)	Strong	Geels 2004 [28] (Netherlands)
Periods of crisis as windows of opportunity for social learning, novelty and experimentation	Strong	Olsson *et al.* 2006 [12] (Sweden)	Moderate	Smith *et al*. 2013 [53] (United Kingdom)
Processes of scaling-out and scaling-up social, and social-ecological innovation	Moderate	Olsson and Galaz 2012 [16] (Sweden)	Moderate	Smith and Raven 2012 [29] (United Kingdom)
The role of structural power and human agency over the enactment of sustainability transformations	Weak	Westley *et al*. 2013 [20] (Canada)	Moderate	Avelino and Rotmans 2009 [44] (The Netherlands)

Olsson *et al*. [7] argue that an integration of the social-ecological and socio-technological systems’ perspectives could help in addressing human-environmental interactions more broadly and that further analysis would benefit from closer collaboration between the fields of resilience and transition management (see Table 1 for an analysis of how these two approaches can strengthen each other). Nevertheless, as we later elaborate, this necessitates a safe space that would allow for reflexive, transparent and inclusive deliberation on current tensions and grey areas as well as to allow researchers to draw from other relevant studies to fill in intellectual gaps. For example, there is still a form of unresolved tension of an ontological nature despite the obvious convergence of the conceptual paradigms in their interpretation of transformation. Where social-ecological systems research is concerned with maintaining a particular flow of services in a given context to achieve a desired outcome, and technology is seen to act on this process, the transitions approach puts technology and social practices at the centre of the analysis, explicitly assuming the existence of interdependent heterogeneous power relationships [42]. However, both appreciate that building transformative capacity and enabling shifts to new trajectories requires systemic experimentation and innovation [7]. The intellectual and experimental potential of combining these two perspectives into a new, emergent way of seeing how sustainability transformations occur could provide a powerful tool for addressing pressing environment and development challenges from the local to the global level.

### 2.4. Emerging Economies: Critiques and Challenges

Many of the largest social-ecological changes are currently happening in emerging economies, which also contain some of the fastest growing populations on the planet with a relatively young age on average. They therefore have a major role to play in addressing global challenges that will also usher in a new 21st century workforce for a meaningful and equitable approach to sustainable development. However, without a good understanding of how these countries are tackling complex sustainability issues across different sectoral domains, it is difficult to imagine how the future will play out globally as well as what pathways to a sustainable future or Good Anthropocene are possible. Until now, this relatively recent topic of social-ecological transformations has largely been formulated and debated by Northern academics working in European and North American universities, including the NeWater project mentioned earlier (see Table 1 where all first authors on these seminal papers are based in North America or Europe). Critical theoretical questions and applied experiments of SETs for emerging economies undergoing rapid social-ecological change are still relatively limited with most work in this area having a development lens, such as the pivotal research coming out of the STEPS Centre and Institute for Development Studies at the University of Sussex, E.g. [54].

An important challenge for emerging economies is to identify what are the enabling conditions required for social-ecological innovations to contribute to systemic social-ecological transformations in these countries. How do these enabling conditions vary across different world regions and social contexts? And how can research and innovation be leveraged through Global South-North as well as South-South collaboration? New constellations of actors and social network configurations are influencing change and guiding processes of innovation and transformation in emerging economies in ways that are often beyond the reach of the state and can be surprising. For example through new kinds of development assistance paradigms such as the use of mobile money platforms to perform cash transfers, or locally based development programmes that are managed directly by citizens [55]. Such initiatives tend to be mobilised by groups of actors that can potentially catalyse and enhance success of SETs in emerging economies through both their collective and individual roles as change agents with capacity to bring together different stakeholders, changing existing power structures or re-directing the flow of resources [20,55]. Countries such as the BRICS nations are currently undergoing processes of democratisation and decentralisation that are creating avenues for innovation both within [56] and outside traditional areas of state influence in particular through the growing relevance of civil society organisations [57]. Ignoring the dynamism and potential of these processes in the global South would severely inhibit the sustainability transformations agenda and yet very little research is seen from this angle.

### 2.5. Emerging Economies: Initiatives that Address Sustainability Transformations

The understanding of social innovations and multi-scale public-private partnerships and investments that support the scalability of high impact sustainability solutions is particularly relevant in emerging country contexts. Including impact investing, crowd-funding, legislative practices and accelerators, incubators and “change-lab” training programs, which borrow from the tech-oriented business sector [58,59,60]. But a critical gap still exists between private sector activity and academic research into the efficacy and impact of these initiatives and the role they might play in fundamentally changing human-environment interactions. These are challenges that are not insurmountable yet do require deeper engagement across emergent perspectives and new theories on transformations. They further necessitate inclusive dialogues and better emphasis on the role of interactive innovation spaces within which stakeholders can co-produce solutions together with transformation scholars, decision makers and private sector stakeholders [61].

There are a series of current initiatives that are relevant to sustainability transformations in emerging economies. Indian academics have been researching the ability of grassroots movements to develop social-ecological innovations for sustainable development, which is being led by Anil Gupta’s Honeybee Network and others in the Inclusive Innovation arena [62,63,64]. This research documents how marginalized villages and urban slums have developed products and services to meet their unmet needs around food, water, energy, education and health systems. In Brazil, policy-focused social innovation initiatives and state support often amalgamate around the notion of a Solidarity Economy [65], which is a value driven conception of the economy. Other scholars have used the national systems of innovation concept to challenge Western interpretations of innovation for development [66], extending the language of innovation to include notions like ‘scarcity-induced innovation’ [67]. In South Africa, the work being done at the University of Cape Town on social innovation that arises from both the private and civil society sectors and that extends to the environmental arena [58] shows a maturing appreciation of new forms of innovation that can deal with the complex challenges facing the country. As an example, there is currently interesting work taking place in Cape Town, such as the R-Labs ‘social revolution’ that aims to empower poor communities through innovation and has spread from South Africa to 22 countries [68]. Many of these initiatives are examples of what has been defined as a Good Anthropocene, and part of scoping plausible “good” futures. The Future Earth fast-track initiative entitled ‘Bright Spots: Seeds of a Good Anthropocene’ project documenting positive experiments /projects/ideas is one such initiative (See the project website at: http://www.pecs-science.org/research/workinggroups/seedsofagoodanthropocene.4.3056027e148c326944337ff.html). The critical aspect of these types of projects is that they ‘open up’ spaces for trans- and inter-disciplinary research that does not close down around specific theoretical approaches as is the case with most other comparative studies. Engaging in theoretical experimentation is difficult in the current academic environment and so it is of vital importance to add legitimacy to this type of research by creating a safe space within which these interactions can take place [69].

## 3. Guiding Principles for Safe Space Experimentation

### 3.1. What is a Safe Space?

A safe space concerns the interdisciplinary development of theories, models, instruments, strategies and impacts that build upon different bodies of knowledge and experience. We find that the research agenda on SETs for sustainability is challenging, but highly societally relevant and thus provides a point of attraction for different theoretical models and disciplines. To this point the notion of safe space has been initially proposed and further developed largely by German sociologist and philosopher Jürgen Habermas. He was one of the first scholars to articulate the notion of the safe space as part of his theory of communicative action. In his account, the safe space, relates to how in the public sphere it is necessary to create conditions for diverse citizens to freely express their views, opinions and beliefs [70]. The idea of a safe space has further gained relevance as part of debates on re-positioning the role of science in society [71] as well as fostering transdisciplinary processes of engagement in sustainability research [72,73,74]. It is in particular in these debates to which we have turned in order to gain deeper insights around its conceptual utility.

### 3.2. Safe Space Principles for Real World Problem-Solving

The safe space lends itself to different interpretations partly depending on the disciplinary domain of use, such as for example sociology, philosophy of science and knowledge as well as the particular problem that a safe space is envisaged to address. In Table 2 we provide a summary account of a range of perspectives that inform our own account of the safe space. Habermas’ account of a safe space is as an emancipatory space in knowledge co-production where a variety of social actors can freely express different views; this is a particularly relevant precondition for other forms of safe space interaction to become subsequently realised. Foremost, we have argued that fostering greater debate between theoretical models such as the ones described under the broad categories of resilience and transitions are relevant to safe space interaction. In these approaches, safe space has also the function of a nurturing space for diverse experimental ideas, transformative innovations and adaptive piloting to be shielded and grow. A safe space is overall a space to freely think without the weight of a disciplinary history or institutional commitments to a given approach that may constrain dialogue, co-create and prepare innovative ideas and interventions. Furthermore, this open space empowers those that have previously not inhabited the sustainability transformations space, especially researchers and practitioners from emerging economies, to be able to engage with, learn from and share their experience with others.

That is why, in a safe space we believe that there is a need to foster reflexivity particularly about how sustainability problems are defined, who is doing the defining, and what are ultimately the main prescriptions for action [42]. Reflexivity is also an underlying principle to ensure that those that operate in the safe space can overcome the innovators’ blindness (seeing only gains and those who gain, having an oversight of losers and conflicts). If this is achieved early on in the process, there will be higher chances for the safe space to succeed as both a platform for knowledge co-production and for fostering transformative learning as well as for invigorating how to democratize inclusive innovation for sustainability [11,75]. Finally we align with Klay *et al.* [76] who propose that safe space formation is likely to proceed in different phases. Starting from a strategically managed safe space (or niche) for multi-stakeholder dialogue that can gain momentum to become gradually a broader thought collective capable of initiating paradigm transformation.

**Table 2:** Principles of the ‘safe space’ advocated in this paper.

Main Focus of the Safe Space	Study Example
***Emancipation and empowerment:*** An emancipatory space in which diverse social actors can freely express different views in favour of communicative action	Habermas, 1981 [70]
***Ensuring reflexivity:*** Broadening reflexive processes of engagement with the definition of sustainability problems, the associated generation of knowledge and prescriptions for action	West *et al*. 2014 [42]
***Knowledge co-creation:*** Active engagement for knowledge (co)-creation through processes that facilitate deep interaction between different stakeholders and (sub)-disciplines	Mitchell and Nicholas 2006 [11] Polk *et al*. 2014 [77]
***Transformative learning:*** Enable learning on how to alter thinking, practicing and organising towards new practice sets and paradigms that can better connect individual action with social change and broader slow and fast social dynamics.	Lange 2004 [75]
***Nurturing innovations:*** Niche creation and management for harnessing ideas and capabilities for the transformation of science	Kläy *et al*. 2014 [76]

These principles are in part informed by our own review of how the safe space is articulated in other disciplines and problem domains (see also previous section) and in part by our own experience as researchers working at the interface of transitions and resilience research in both North and South contexts. Therefore the principles are particularly tailored to trigger and accelerate dialogues and interactions across these two perspectives specifically. While of course recognising that accelerating transformations is not a transitions or resilience task *per se*, and that it also involves insights and learning from other disciplines and practice orientated learning environments. Finally, we aim to remain critical and further reflect on which type of epistemological pathways we can envision from the ‘safe space’ perspective we have outlined in this section.

### 3.3. Deepening Engagement with Contexts of Inequality and Exclusion

Current debates and scholarship on transformations for sustainability still miss substantive insights on the complex interactions of both formal and informal interventions within contexts of inequality, poverty and social discontent. In order to move forward with a sustainability agenda, the safe space can be envisaged as a platform to provide direction on how to confront directly the complexities of inclusion and exclusion and to address current power asymmetries when co-designing transformational pathways.

Specific issues of culture, class-based discrimination and national identity formation may have a major role to play in the formulation of sustainable transformations. Who decides to initiate transformations? Can transformations be carried out in a deliberative, participatory manner that is both ethical and sustainable? How can power, politics, and interests present barriers, or pathways, to transformation? These are questions raised by O’Brien [8] that have received only limited attention in existing transformations scholarship, but are particularly relevant in developing country contexts not least because of growing concern about inequality along various indicators (such as wealth, access to basic services and employment) [78,79,80,81]. The emergence of the Digital Divide is a relevant case study. In trying to expand market accessibility to impoverished and isolated communities, Mobile Network Operators (MNOs) have extended digital connectivity to many remote areas [82]. However, instead of opening up the information economy to all within reach, the majority of usage is by wealthier and privileged populations due to cultural, language, literacy and financial barriers as well as limited access to information and communication technologies (ICTs). These populations then use these new information and social network resources to further their own prosperity and exacerbate existing social inequalities, thus developing a Digital Divide. Recent work focusing on new technologies for information generation in the environmental management field, identifies significant scope and potential for making science and data more transparent and accessible to citizens while new technologies can also serve as platforms for facilitating deep multi-actor collaboration and learning [83].

Such examples of power dynamics, often around gender, have been raised mainly by political economy researchers who have critiqued resilience thinking as being an insufficient means of engaging in the sustainability debate in developing country contexts [25,84]. Researchers working on agricultural adaptation to climate change in Tanzania, have proposed a middle-ground, arguing for a combinations of ‘resilience thinking’, ‘political ecology’, and environmental anthropology as a way of embedding analysis of power struggles and cultural norms in the context of the overall socio-ecological system [85]. However, it is not simply a theoretical argument, but fundamental methodological challenges arise in bringing these approaches together, as well as difficulties in how findings can be applied to contexts where practitioners are not experienced with these theoretical approaches [86]. The safe space advocated for in this paper would provide a legitimate home for experimentation between these different theoretical approaches, with the ultimate aim of developing a coherent set of approaches that would be relevant to the specific challenges in developing world contexts.

## 4. Discussion and Conclusions

In this article we have drawn on the notion of a *safe space* to elucidate possibilities for multi-stakeholder learning and collaboration. Specifically, we have drawn the main insights from two important inter-disciplinary research strands that have come of age in understanding transformations to sustainability: sustainability transitions and resilience.

We have illustrated the potential for synergy that can emerge by focusing on greater collaboration between these topic areas, but other disciplinary perspectives, despite not explicitly dealing with transformations, can play an important role in the future design of safe spaces. A particularly important strength of resilience and transitions, is that they can support a better understanding of how ideas and innovations spread in society, placing particular emphasis on those ideas and innovations that can help tackle complex real-world problems from economic development to healthcare [6,22]. We find that there is tremendous scope for enhancing a more collaborative research environment that can bridge impact-oriented action researchers and reflexive practitioners who are currently engaged in facilitating SETs in different contexts, in the global North and South. It should be noted that there is a need to recognize the subjectivity that researchers bring along with their epistemological frameworks and scholarships, and to aid a deep reflection on the way these frameworks are applied or enforced through empirical research in the South. This paper argues for a more open and transparent way of doing research and co-producing solutions where assumptions and research hypotheses remain open to change and open to dialogue not only between academics but also between research practitioners and stakeholders from vastly different backgrounds. Without comparative research being seen as a treatment to this problem, more careful research design that will also enable researchers to break free from epistemological subjectivities and move towards research experimentation will further the ‘safe space’ to be embedded in practice globally.

The safe space is essentially a collaborative environment and therefore more than just a ‘marriage’ of resilience and transitions research. It is more fundamentally about recognising the opportunities associated with pluralising knowledge systems [87] as a stepping stone towards enacting SETs. We have further argued that perhaps the most exciting arena for the safe space to flourish is in emerging economies where the issue of transformation is likely to be more fundamentally not only about the deliberation and enactment of ‘sustainable’ transformations, but also equally about ‘just’ transformations based on greater community understanding, equality and justice [88].

Acknowledging the opportunity context that the need for systemic transformations offer, while also allowing for reflexivity and transparency in collective processes of imagining and enacting sustainability pathways, is a key aspect of the ‘safe space’ approach advocated in this paper. This is why the paradoxes, tensions and ‘grey areas’ that are shaping debates on transformative change and how these are enacted differently across academia, civil society, public and private settings have to be made explicit in navigating the safe space—without them there would be no need to experiment beyond conventional approaches. We are also aware that there is a danger that the safe space could lead to consensus frames that are depoliticized and lack the necessary substance to allow for concrete action and provide direction for SETs [89]. It is essential to remain attentive to the issue of disparity, which is the manner and degree in which categories will tend to differ from one another [90,91]. Engaging with these disparities in a constructive manner will critically depend on the extent to which the safe space can allow for analytical communities to emerge that can balance between pluralism and concreteness in an effective and durable way [90]. There will always be aspects of each knowledge system that cannot be fully translated into each other, partly because of the different approaches that exist (within disciplines and across different knowledge systems) [92,93] and this is actually positive for the safe space because it is not designed to be a replacement for existing approaches. What we have set out in this paper is not a replacement viewpoint, but an alternative means through which to navigate the planet’s need to transform both sustainably and justly.
